# Initiative on Superselective Conventional Transarterial Chemoembolization Results (INSPIRE)

**DOI:** 10.1007/s00270-022-03233-9

**Published:** 2022-08-17

**Authors:** Thierry de Baere, Maxime Ronot, Jin Wook Chung, Rita Golfieri, Roman Kloeckner, Joong-Won Park, Bernhard Gebauer, Nabil Kibriya, Ganapathy Ananthakrishnan, Shiro Miyayama

**Affiliations:** 1 Institut Gustave Roussy, Service Radiodiagnostic et Imagerie Médicale, 39, rue Camille Desmoulins, 94800 Villejuif, France; 2 Department of Medical Imaging, Beaujon University Hospital, Clichy, France; 3 Department of Radiology, Seoul National University College of Medicine, Seoul, South Korea; 4 Unità Operativa Radiologia Universitaria (Pad 1, 2), Dipartimento delle Radiologie, IRCCS Azienda Ospedaliero-Universitaria di Bologna, Policlinico S. Orsola-Malpighi, Bologna, Italy; 5 Radiology Department, Mainz University: Johannes Gutenberg Universitat Mainz, Mainz, Germany; 6 Center for Liver and Pancreatobiliary Cancer, National Cancer Center (NCC), Goyang-si, South Korea; 7 Department of Diagnostic Radiology, Charité Universitätsmedizin Berlin, Campus Virchow-Klinikum: Charite, Berlin, Germany; 8 Department of Radiology, Kings College Hospital, NHS Foundation Trust, London, UK; 9 Department of Radiology, Manchester Royal Infirmary, Manchester, UK; 10 Department of Diagnostic Radiology, Fukui-Ken Saiseikai Hospital, Fukui, Japan

**Keywords:** Hepatocellular carcinoma, Conventional transarterial chemoembolization, Iodized oil, Selective embolization

## Abstract

Several publications show that superselective conventional TransArterial ChemoEmbolization (cTACE), meaning cTACE performed selectively with a microcatheter positioned as close as possible to the tumor, improves outcomes, maximizing the anti-tumoral effect and minimizing the collateral damages of the surrounding liver parenchyma. Recent recommendations coming from the European Association for the Study of the Liver (EASL) and European Society of Medical Oncology (ESMO) highlighted that TACE must be used in Hepatocellular Carcinoma (HCC) “*selectively targetable*” and “*accessible to supraselective catheterization.*” The goal of the manuscript is to better define such population and to standardize superselective cTACE (ss-cTACE) technique. An expert panel with extensive clinical-procedural experience in TACE, have come together in a virtual meeting to generate recommendations and express their consensus. Experts recommend that anytime cTACE is proposed, it should be ss-cTACE, preferably with a 1.5–2.0 Fr microcatheter. Ideally, ss-cTACE should be proposed to patients with less than five lesions and a maximum number of two segments involved, with largest tumor smaller than 5 cm. Angio Cone-Beam Computed Tomography (CBCT) should be used to detect enhancing tumors, tumor feeders and guide tumor targeting. Whole tumor volume should be covered to obtain the best response. Adding peritumoral margins is encouraged but not mandatory. The treatment should involve a water-in-oil emulsion, whose quality is assessable with the “drop test.” Additional particulate embolization should be systematically performed, as per definition of cTACE procedure. Non-contrast CBCT or Multi-Detector Computed Tomography (MDCT) combined with angiography has been considered the gold standard for imaging during TACE, and should be used to assess tumor coverage during the procedure. Experts convene that superselectivity decreases incidence of adverse effects and improves tolerance. Experts recommend contrast-enhanced Computed Tomography (CT) as initial imaging on first follow-up after ss-cTACE, and Magnetic Resonance Imaging (MRI) if remaining tumor viability cannot be confidently assessed on CT. If no response is obtained after two ss-cTACE sessions within six months, patient must be considered unsuitable for TACE and proposed for alternative therapy. Patients are best served by multidisciplinary decision-making, and Interventional Radiologists should take an active role in patient selection, treatment allocation, and post-procedural care.

## Introduction

Hepatocellular Carcinoma (HCC) is the most common primary liver cancer, considered the sixth leading cause of cancer and the third leading cause of cancer-related mortality [1, 2]. In 2020, liver cancer (predominantly HCC) accounted for over 830,180 deaths, with an estimated incidence of 905,677 cases; mortality rates being particularly high in Asia [2]. HCC most often develops in patients with chronic liver disease, including hepatitis B and C, alcohol abuse, and non-alcoholic fatty liver disease [1, 3, 4].

The general treatment approach is guided by the internationally agreed Barcelona Clinic Liver Cancer (BCLC) treatment algorithm, with treatment options including surgery, liver transplantation, ablation, radiotherapy (including external and selective internal radiotherapy), transarterial chemoembolization (TACE) and systemic therapy [5]. A multi-disciplinary approach is considered key for treatment decision according to individual condition [6].

While the BCLC is the standard guideline for the US and Europe, it is important to consider that there are other staging and treatment allocation systems, such as the Japan Integrated Staging (JIS) scoring system, the Chinese University Prognostic Index (CUPI), or the more recent Hong Kong Liver Cancer (HKLC) staging system that are driving Asian practice. In general, these favor a more extensive use of local therapies in terms of treatment allocation when compared to BCLC [7–11], and although well known, have not yet been clearly validated in Western populations.

In most, if not all, current staging systems, TACE is the recommended therapeutic option for asymptomatic patients with liver-only limited tumor burden without macrovascular invasion (MVI), who are not amenable to surgery or ablation. TACE treatments aim at inducing tumor necrosis and are based on the predominant arterial vascularization of HCC compared with the surrounding liver parenchyma [12, 13].

The role of TACE is challenged by constant developments of other treatments. According to the recent update of the BCLC staging system, systemic therapy, including immune-oncology, is recommended for patients with intermediate-stage HCC harboring diffuse, infiltrative, extensive bilobar liver involvement [5]. Additionally, TACE might be combined with systemic therapy in an effort of TACE debulking, or TACE as an enhancer of immunotherapy by tumor antigen release [14]. This moving landscape calls for a clear reappraisal of what is an optimal TACE, as well as suitable identification of patients amenable to superselective TACE (ss-TACE).

Historically, conventional TACE (cTACE), through the intra-arterial delivery of anticancer drugs emulsified in Lipiodol, has been the well-established treatment for patients with intermediate-stage HCC [10], with a high level of evidence and strong recommendation following two randomized controlled trials (RCTs) released in 2002 [15, 16] and two decades of practice [17]. In fact, over the past twenty years following the publication of these seminal trials, the outcome of cTACE has improved [18]. Such improvements are likely explained by better patient selection and cTACE technique refinement. The former is illustrated by the most recent BCLC update that limits TACE indication to patients who should be selectively targetable [18]. The technical refinements, on the other hand, mainly include: standardization of water-in-oil Lipiodol/drug emulsion preparation in specific ratios, which maximize the propensity to target the tumor feeding arteries [19], improvement of imaging quality during fluoroscopy or digital subtracted angiography, and advanced 3D imaging technology available during treatment delivery.

Several publications suggest that cTACE performed selectively improves outcomes. In fact one of the most significant progresses regarding cTACE technique within the last decade has been superselective cTACE (ss-cTACE). This means positioning the catheter as distal as possible and close to the tumor. Strong evidence [12, 20] shows that ss-cTACE maximizes the anti-tumoral effect and minimizes the collateral damages of the surrounding liver parenchyma, thus optimizing the therapeutic effect on HCCs. Yamakado et al. have reported that technique has an impact on patient survival, in an HCC population of ≤ 7 cm nodules and with ≤ 5 lesions: the prognosis of patients who underwent selective/superselective cTACE was significantly better than that of patients treated with non-selective cTACE (*p* = 0.033) [21].

However, while many operators regularly use this term to describe their routine procedures, there is still much to be understood and defined regarding rationale, patient selection, and all technical aspects of ss-cTACE.

To this purpose, and to validate the best current practice for ss-cTACE, a panel of international expert physicians took part in a virtual consensus meeting on May 29th, 2021, to draft common recommendations based on accumulated data and practices and ultimately to standardize ss-cTACE treatment in HCC. All experts have extensive experience in TACE (ranging from a minimum of 11–to 35 years to date), most of whom work in leading international centers of excellence. The selection was purposely heterogeneous to reflect different standards and practices throughout Europe and Asia. In particular, the board included physicians from France (*n* = 2), Germany (*n* = 2), Italy (*n* = 1), UK (*n* = 2), Korea (*n* = 2), and Japan (*n* = 1). Different topics were discussed as summarized below, and the recommendations were collaboratively generated during the virtual meeting. The lead author wrote the following manuscript that was edited and approved by the group before submission.

## Patient Selection

All experts agreed that cTACE should be performed superselectively whenever possible, since it has shown to increase efficacy on the tumor [13, 20, 22] and limit toxicity to non-tumor liver parenchyma [23]. As reported in a paper by Craig et al., which clearly shows the lack of standardization and common practice in the daily IR routine [24], and confirmed by Tsochatzis et al., TACE remains an unstandardized procedure [25], which according to experts is often not performed in a superselective manner. In fact, ss-cTACE is possible in a subset of HCC patients only, namely those with limited tumor load regarding tumor size and number. According to the literature and as agreed upon by the panel, ss-cTACE is highly feasible and particularly useful in tumors of a maximum diameter up to 5 cm (and possibly up to 7 cm) [21, 26]. In this setting, most treatments can be performed in a single session, and the reported complete response (CR) rate according to modified Response Evaluation Criteria in Solid Tumors (mRECIST), ranges between 42 and 91% [20, 27–31], according to patient selection and nodule dimensions.

In regards to the ideal patient who could benefit from a superselective approach, this is clearly also linked to the size of the lesions treated. In a study on cTACE efficacy through pathological analysis of the whole lesions after liver transplantation, a complete histological necrosis was achieved in 53.8% of lesions after selective/superselective TACE: 91.8% after ss-cTACE versus 66.5% after lobar procedures [20]; and in particular necrosis was maximal for tumors > 3 cm. Larger tumors are less likely to be amenable to ss-cTACE due to the multiplicity of tumor feeders, higher dose of drug and Lipiodol required, and the need for several sessions to obtain the best response possible. In another study, a CR after the first cTACE was achieved in 68% in < 2 cm HCCs; 64% in 2.1–5 cm HCCs and 25% in > 5 cm HCCs [32].

Furthermore, HCC lesions greater than 3 cm are often associated with the need for multiple treatments, and tumor involvement > 50% of the liver volume is associated with a poor prognosis [20].

The number and location of tumors (and the subsequent number of segments involved) are apparent limitations that can render a patient suboptimal or even completely unsuitable for ss-cTACE due to the evident possibility of widespread disease. Therefore, experts agreed that ss-cTACE should be recommended for patients with less than five lesions and a maximum number of two (and possibly up to four) segments involved. The size of the largest tumor nodule is often considered most important in patients with an oligo-nodular disease. Interestingly, a recent publication highlighted the significance of the smallest size tumors that could or not coexist with a large tumor. This seems to imply that the smaller the tumor, the higher the probability for undetectable lesions to be present [33]. Consequently, very small sized tumors must be considered in the treatment decision algorithm.

In an attempt to further standardize TACE procedures, it is worth noting that specifically for the population defined above as being optimal for ss-cTACE, cTACE demonstrated better outcome than drug-eluting beads (DEB-TACE) in a very recently published RCT. Indeed, 200 Child–Pugh class A or B patients with unresectable and treatment naïve HCC (minimum diameter of 10 mm and maximum diameter of 50 mm), scheduled to receive selective TACE, were randomly assigned at a 1:1 ratio to DEB-TACE or cTACE. The study was positive for the primary endpoint (CR rate at 3 months) with 27.6% in the DEB-TACE arm and 75.2% in the cTACE arm (*p* < 0.0001) [34].

Of note, a significant subset of patients included in the two 2002 pivotal randomized studies [15, 16] that validated TACE as the standard of care, had limited tumor burden. In the study by Lo et al., 43% of patients had a single tumor, and in that by Llovet et al., 50% of the population had one or two tumors. The mean diameter of the largest tumor was 49 mm in the former trial, with no tumor larger than 60 mm. While these studies came early in establishing the basis of the best candidate for TACE as discussed here, during the following years the spectrum of patients was broadened, ultimately leading to considerable heterogeneity among published studies. In response, many publications tried to refine the BCLC B stage, using various criteria such as quasi C [35] or up-to-7 [36]. Noteworthy, the most recent guidelines in Europe acknowledge that TACE should be restricted to patients amenable to superselective TACE, as defined in the updated 2021 ESMO guidelines, on HCC “accessible to supraselective catheterization” [37], and with “selective access,” in the recent update of the BCLC strategy for prognosis prediction and treatment recommendation [5].

In conclusion to this topic, even if ss-cTACE continues to generate promising results with recognized curative potential, it remains clear that surgery and thermal ablation have superior benefits when feasible and should remain the treatment of choice in such cases. Consequently, small tumors should be proposed to ss-cTACE only in the scenario of treatment stage migration or when patients are enlisted for a liver transplant.

The expert panel recommends

 − That anytime cTACE is proposed, it should aim at being ss-cTACE.
 − That ss-cTACE should be ideally proposed to patients with tumors smaller than 5 cm (and possibly up to 7 cm), aiming at a complete response.
 − That ss-cTACE should be recommended for patients with less than five lesions and a maximum number of two segments involved

## Treatment Delivery

TACE is considered superselective if delivered at a subsegmental level by a microcatheter reaching the tumor feeders through terminal and intersegmental collaterals [12], while sparing the surrounding healthy liver parenchyma as much as possible. However, the need to treat a limited volume of healthy liver around the target tumor in order to achieve safety margins was highly debated among experts, with difficulties to obtain a consensus. In a study by Miyayama et al., including 102 patients with up to 5 tumors (each measuring up to 7 cm) treated by cTACE, 82.1% of tumors were completely embolized with a safety margin, 13.7% had complete tumor embolization without a safety margin, and 4.2% had incomplete embolization. The 1-, 3-, and 5-year local tumor progression (LTP) rates were 31.7, 49.4, and 59.4%, respectively. LTP developed less frequently in tumors treated with a 5-mm safety margin for tumors < 25 mm, and 10-mm wide for tumors 25–60 mm (*p* = 0.0016). In addition, LTP has been reported to be linked with intrahepatic distant progression [38]. Despite many other studies repeatedly highlighting the benefit of including peritumoral margin when performing ss-cTACE [39–42], not all experts in the panel agreed on the need and the extent of safety margins, even if they are predictive of success, rather converging on the statement that complete lipiodolization of the tumor with a clear margin should be obtained. The reason for this absence of consensus is that when compared with percutaneous thermal ablation, it is more difficult to achieve complete safety margins through an intraarterial approach, and achieving such safety margin would require to “sacrifice” a larger volume of normal liver parenchyma. However, there was unanimous agreement that the treatment must cover the whole tumor volume to avoid any defect in Lipiodol uptake in targeted tumors that have been confirmed to negatively correlate with tumor response [43].

Regarding general procedural quality and success, experts agreed that excellent imaging quality during the procedure is critical to optimize guidance, to ensure appropriate targeting of the tumor and to guarantee high standards and reliable post-treatment assessment. Consequently, either CBCT angiography, or MDCT-angiography are considered mandatory to improve precise tumor detection, local staging, targeting, and preserve as much healthy liver as possible [44]. In a study including 207 HCC patients treated with ss-TACE with angio-CBCT (CBCT, *n* = 109) or with digital subtraction angiography (DSA, *n* = 98), significant differences were reported between both groups in terms of technical success (*p* < 0.001). The 1-, 2-, and 3-year local recurrence rates were 33.3% vs. 22.3%, 41.3% vs. 26.8%, and 48 vs. 30.6%, in the DSA and angio-CBCT groups, respectively (p = 0.0217) [45]. In another study including 45 patients with 66 hyper-enhancing tumors (mean 32 mm ± 18, range 10–81 mm) treated with TACE, angio-CBCT was analyzed with and without dedicated software to identify tumor-feeders. Among 179 feeders, the sensitivity of the software was significantly higher than that of conventional analysis (90.9% vs. 82.1%; *p* < 0.0001), with lower positive predictive value (82.9% vs. 90.6%, *p* < 0.0001), higher false-positive ratio (17.1% vs. 8.8%, respectively; *p* < 0.0001), and greater inter-reader agreement (92% vs. 79%, respectively; *p* < 0.0001) [46]. Furthermore, next-generation virtual parenchymal perfusion software using angio-CBCT, allowing pre-treatment prediction of complete target coverage, was recently evaluated in 56 patients (91 tumors) treated by TACE. Authors reported a mean Dice similarity coefficient of 0.78 ± 0.01 between actual and virtual embolized areas, with good correlations between volumes (*r* = 0.957, *p* < 0.001) and a mean surface distance of 2.78 ± 2.11 mm [47].

Therefore, as a final comment regarding the imaging tools required for an optimal procedure, all experts agreed on the value of guidance software to extract tumor feeders from a whole liver CBCT or MDCT angiograms to identify all tumor feeders and then target the whole tumor volume.

Regarding the most suitable and appropriate microcatheter for the procedures, 2.4 French (F) was recognized as the upper caliber limit accepted to perform ss-cTACE. However, all experts agreed that lower microcatheter diameter, down to 1.5–2.0 F, shall be preferred and recommended when possible. In fact, smaller caliber microcatheters are required to increase likeliness of reaching more distal target injection points, improving tumor targeting, and minimizing non-tumor liver damage. Further consensus was reached that once positioned in the tumor feeder, the microcatheter does not need to be wedged but that a semi-wedge positioning is acceptable. Similarly, following the Lipiodol/drug emulsion delivery, the microcatheter should be slightly pulled back to a more proximal position for final embolization.

Another important topic that is often object of discussion across centers and varies according to local preference, is the most appropriate drug to use for cTACE. Chemotherapy regimen selection varies from center to center, from East to West, and there is great heterogeneity in the number of drugs and their combinations used for cTACE, also based on the different availabilities in various countries. Doxorubicin, Epirubicin, Idarubicin, and Cisplatin are the most commonly used singularly, while the most common combination regimen is doxorubicin and cisplatin [17], as acknowledged by the expert panel. Nevertheless, there are papers showing potential benefits of multi-drug regimens, and that other drugs such as mitomycin C, hydroxycamptothecin, fluorouracil, serve as alternative agents [48–50], but the scenarios and indications for such alternatives have yet to be determined. Also, the results reported are mainly referable to large tumors up to 10 cm, and for this reason not necessarily relevant to the population we consider as ideal for ss-cTACE.

In regards to single-drug treatment in particular, no specific chemotherapeutic product has demonstrated superiority over another. In a recent study, 455 patients were randomly assigned to undergo TACE with cisplatin (*n* = 228) or epirubicin (*n* = 227) resulting in a median overall survival (OS) of 2.93 years (95% CI 2.60–3.79) and 2.74 years (95% CI 2.26–3.21), respectively (hazard ratio 0.90 [95% CI 0.71–1.15], *p* = 0.22) [51]. Similarly, a study comparing cTACE with miriplatin vs. cTACE with epirubicin showed no significant difference in median OS and median times to TACE failure between the two groups, proving once again no superiority of one single drug over another [68].

Another topic on which there was full consensus, was the importance of the oil and cytotoxic drug mixing, even though from the survey published by Craig et al. respondents stated not to have a preferential method of mixing (249/505, 49.3%) [24]. In particular, it has been widely demonstrated that a water-in-oil emulsion made of droplets (i.e., an internal phase containing drug in aqueous solution and a continuous external phase of oily Lipiodol) is more densely retained within the tumors than the alternative oil-in-water emulsion [52]. In order to obtain the proper water-in-oil emulsion, which also has a greater propensity for drug delivery to the tumor, a specific ratio between drug and Lipiodol volumes should be respected: ideally one volume of drug to two or three of Lipiodol (water/oil = 1:2, 1:3).

In an experimental study [53] aiming to compare water-in-oil (W/O) emulsion ss-cTACE followed by gelatin particles and bland microspheres in transarterial embolization (TAE), using a rat hepatocellular carcinoma model, the percentage of necrotic area and complete response ratio in the W/O emulsion group was significantly higher (99.9 vs. 87.6%, *p* = 0.029 and 87.5 vs. 28.6%, *p* = 0.041, respectively). These results showed stronger antitumor effects with the occlusion of both the tumor feeding artery and the portal vein compared with microspheres, which occluded only the arteries. Serum aspartate aminotransferase (AST) and serum alanine aminotransferase levels (ALT) 48 h after treatments were significantly higher in the W/O emulsion group (*p* < 0.01), This increase testifies what has been demonstrated that post-treatment transient transaminase elevation is predictive of objective response to ss-cTACE [54], since transaminases are produced by both hepatocytes and hepatocyte-derived tumor cells. Additionally, considering the lack of liver functional reserve deterioration, it is conceivable that the serum concentration elevation of these enzymes is of tumor origin, thus justifying the correlation with tumor response.

Using a non-ionic contrast medium to prepare drug aqueous solution increases the density of the drug solution by lowering the sedimentation process induced by gravity, thus favoring emulsion stability [55]. The emulsion is prepared using Lipiodol resistant 3- or 4-way stop-cocks and syringes that are not damageable by the emulsion. The content of the syringe containing the drug should be first pushed towards the syringe containing Lipiodol, in order to initiate the water-in-oil emulsion, initially with large drops of drug within Lipiodol. Vigorous mixing of the chemotherapy aqueous solution and Lipiodol via the adequate stopcock generates sufficient energy to decrease the size of the internal phase droplets. It has been tested that at least 20 pumping exchanges through the stopcock are needed to obtain an internal phase size of droplets in the range of 70–100 microns [19], although it has been demonstrated that the mean droplet diameter decreases non-significantly when the number of pumping exchanges exceeds 20, and increases significantly over time, with the droplets returning to their initial diameters after re-mixing [56].

Furthermore, incremental addition of 2 to 4 aliquots of the drug to the full Lipiodol volume, or even continuous addition, results in a highly predictable water-in-oil emulsion type and a significant increase in stability compared to single bolus injection [57]. The incremental addition method may become even easier and user-friendly when possible to rely on dedicated Lipiodol resistant devices, ideally incorporating components such as a 4-way stopcock, or a glass pumping emulsification device [58]. The emulsion must be prepared at the time of administration and must be used promptly after preparation. If necessary, the mixture can be re-homogenized during the treatment session. The volume of Lipiodol to be injected is directly related to the volume of tumors to be treated. A standard dose of up to 10 mL is most often reported in clinical studies and daily practice. Higher volume raises the risk of adverse events, including liver failure, pulmonary toxicity secondary to hepatovenous shunting, and cerebral embolism [59].

Furthermore, once the emulsion is ready to be injected, it is recommended to check for its stability and proper mixing direction. An easily available and reliable way to check direction of an emulsion is the “drop test” [60]. The test consists in pushing a droplet out of the syringe within saline or any aqueous liquid. If the water-in-oil emulsion is correct, the droplet keeps its shape, and the drug remains trapped in the oil, without mixing or “breaking out” into the saline, due to the non-aqueous miscible oily external phase. Conversely, an oil-in-water emulsion droplet will dissolve in the saline, as the external water phase mixes within it, creating a cloudy solution.

Finally, additional embolization must be performed after the drug/Lipiodol emulsion injection, as reported in the 2016 expert recommendation paper by de Baere et al. [61]. This final step is mandatory. Embolization has been reported to increase the rate of necrosis of the main tumor from 13 to 83% and satellite nodules from 6 to 53% [62]. It has also been shown to significantly increase OS, as proven in a series of 11,030 patients including 8057 cTACE and 2523 chemotherapy-Lipiodol injections without embolization [63]. Gelatine sponge particles are the most commonly used embolic material and are associated with arterial recanalization within 1–2 weeks after embolization [64]. This allows for subsequent TACE retreatment through the same tumor feeders. The hand cutting of gelatine sponge particles measuring 1–1.5 mm is the standard practice [65], but recently pre-calibrated gelatin pledgets have become available. Non-resorbable calibrated microparticles have not demonstrated added benefit and could be considered a possible downside due to the permanent occlusion of tumor feeders.

Despite the increasing number of studies endorsing the above-mentioned technical aspects for cTACE, experts agreed that many gaps in their worldwide receptivity and implementation remain, explaining why cTACE technique standardization is still far from solid.

The expert panel recommends

 − That the whole tumor volume should be covered during ss-cTACE to obtain the best response possible. Adding peritumoral margins is encouraged but is not considered mandatory.
 − That tumor coverage should be assessed immediately after treatment with non-contrast CBCT.
 − That angio-CBCT should be used to detect enhancing tumors, tumor feeders and to guide tumor targeting. The use of navigation or simulation software is encouraged.
 − That 2.4 French is recognized as the upper caliber limit accepted to perform ss-cTACE, but lower microcatheter caliber, down to 1.5–2.0 F, is preferred and recommended.
 − That a semi-wedge positioning of the catheter in the feeders is acceptable. The microcatheter should be slightly pulled back to a more proximal position for final additional embolization.
 − That the treatment should be injected as a water-in-oil emulsion of 70–100 microns droplets. To achieve this, the emulsion should be prepared with a lipiodol/drug ratio of 2 or 3 to 1, using lipiodol resistant 3- or 4-way stopcocks and syringes. The use of dedicated preparation devices is encouraged. At least 20 pumping exchanges through the stopcock should be done, and the incremental method be favored.
 − That the quality of the emulsion should be assessed with the ‘drop test’.
 − That the emulsion should be prepared at the time of administration and be used promptly after preparation.
 − That additional feeder embolization should be systematically performed after emulsion injection. Hand-cut gelatine sponge is favored.

## Expected Outcomes

### Efficacy

Since the seminal RCTs by Lo and Llovet comparing TACE to best supportive care [15, 16], the control arms of two more recent large randomized studies evaluating cTACE versus cTACE plus Brivanib or cTACE plus Orantinib can be seen as milestones for cTACE, with reported median OS over 2 years, namely 26.1 months [36], and 32.3 months [66], respectively. More recently, cTACE has reached the 3-year OS barrier with both Miriplatin and Epirubicin, with a reported median OS of 36.5 and 37.1 months, respectively [67]. In addition, a pooled analysis of more than 500 patients also reported up to 35.9 months median OS [26]. This data suggests that a median OS of 25–30 months is probably a valid goal to consider when discussing and predicting ss-cTACE outcomes. This is in line with the expected 2.5-year OS for intermediate HCC according to the 2018 version of the EASL Clinical Practice Guidelines: management of hepatocellular carcinoma [10] and the 2021 update the BCLC staging system [5].

### Safety

Another important aspect addressed during the meeting was related to safety. Superselectivity will likely decrease the occurrence of adverse effects and improve tolerance. In a study including 52 patients with high risk for TACE, ss-cTACE could be performed in 56.9%, and lobar cTACE was performed in the remaining 43.1% of patients. Patients with lobar embolization and multifocal disease had significantly higher mortality rates (*p* = 0.03) [68]. This was confirmed by another study comparing three TACE techniques for the treatment of HCC in 184 patients: non-selective lipiodol-chemotherapy + non-selective embolization (group 1), non-selective lipiodol-chemotherapy + selective embolization (group 2), and selective lipiodol-chemotherapy + selective embolization (group 3). The rate of patients with poor clinical tolerance was lower in group 3 (27.0%) than in group 1 (64.1%, *p* < 0.001) or 2 (66.7%, *p* < 0.001). The rate of patients with poor liver tolerance was higher in group 2 (34.0%) than in groups 1 (17.6%, *p* = 0.050) or 3 (6.9%, *p* = 0.011). The rate of patients with tumor response was also higher when embolization was selective versus non-selective, i.e., group 2 + 3 (78.7%) versus group 1 (62.5%, *p* = 0.054) [69]. Finally, multivariate analysis identified the TACE-technique as an independent prognostic factor for poor clinical tolerance, liver tolerance, and tumor response.

The expert panel

 − Suggests that a median OS of 25–30 months is probably a valid goal to consider after ss-cTACE.
 − Recommends that tumors amenable to ablation should be proposed to ss-cTACE only in the scenario of treatment stage migration or when patients are enlisted for a liver transplant.
 − Stresses that superselectivity decreases the incidence of adverse effects and improves tolerance.

## Post-Treatment Care and Imaging Follow-Up

Post-treatment evaluation starts in the angiographic suite with the help of immediate post-treatment MDCT or CBCT. The first goal is to check for tumor targeting, which can be easily evaluated due to the marked attenuation of Lipiodol. Tumor coverage should be assessed immediately after treatment with CBCT because the lipiodol deposition pattern on immediate post-treatment CBCT images has been shown to closely parallel that of multidetector MDCT performed at early visit after treatment [44]. Furthermore, Lipiodol devoid areas may either denote tumor necrosis or untreated viable tissue, hence multiphase CT is generally recommended at 4 weeks post cTACE, so that Lipiodol can get localized within the tumor [70]

Such assessment ensures that a possible absence of tumor response is not due to inadequate tumor targeting, but mainly to the tumor biology not reacting to the delivered therapy. In addition, the dense tumor staining with Lipiodol on post-cTACE CT is associated with tumor necrosis [71], and Lipiodol deposition has been demonstrated as an early radiological marker for survival [72]. Therefore, the absence of deposition of Lipiodol in parts of the targeted tumors suggests that tumor feeders may have been missed. All panel members acknowledged that the attenuation of Lipiodol is deemed, especially helpful in determining tumor targeting and could serve as a biomarker of tumor response [43].

Post ss-cTACE imaging follow-up requires contrast-enhanced cross-sectional imaging. There is weak evidence for the superiority of MRI with extracellular contrast material versus CT. Experts agreed that MDCT is recommended as initial imaging on the first follow-up after ss-cTACE. If CT depicts a viable tumor, then MRI is not needed. Consequently, CT is recommended as a “triage tool” to select patients likely to have disease control (partial response or stable disease) or progressive disease according to arterial enhancement-based response evaluation criteria (e.g., mRECIST), and for which either a subsequent TACE or second-line treatment is needed. In case of complete mRECIST response at CT, MRI may be used for confirmation and favored during further follow-up to search for possible local progression. MRI is primarily recommended when the complete response on CT seems doubtful, namely in patients without tumor enhancement and with a patchy lipiodol deposition that may mask underneath viable tumors. Indeed, CT can hardly evaluate contrast uptake between or close to the Lipiodol deposition due to its high attenuation [73, 74]. This may impair the assessment of residual or recurrent tumors in their close vicinity.

Another critical aspect discussed during the expert meeting was the timing of subsequent TACE. Unfortunately, the choice of when to stop and retreat is hardly addressed by any guideline. The Assessment for Retreatment with Transarterial chemoembolization (ART) score was developed in the attempt to identify patients who are unlikely to benefit from further TACE sessions [75], but many controversial opinions have arisen on the ART-score, and additional validation was recommended by several groups [76–80].

The goal of ss-cTACE is to obtain a complete response on imaging and, ideally, on pathology. Complete lipiodol uptake is an acceptable endpoint to stop TACE even if obtained after a single session. As mentioned above, MRI may be used to confirm the complete response. In case of disease control or technical failure in targeting one or several tumors, a second session of TACE should be performed to obtain a complete response. Ideally, the second TACE should be scheduled within 3 to 4 weeks—up to 10 weeks—following the first TACE, depending on the tolerance of the first treatment and patient conditions, including liver function and general status. All experts agreed that if no response is obtained after two TACE performed within six months, and in the absence of obvious reasons for inadequate response (e.g., parasitized extrahepatic vessels), the patient must be considered TACE-refractory and proposed alternative therapy. On the other hand, if an objective response is observed, TACE may be repeated more than twice, if needed. Other situations defining patients who could become TACE unsuitable include deterioration of liver function, or patient’s general conditions that can be linked with treatment toxicity or tumor progression. Additionally, TACE unsuitable patients include some type of tumor progression (e.g., extrahepatic, MVI, widespread hepatic progression), compromised arterial access to the tumor to be treated (impaired hepatic arteries, distal vasculitis, uncatheterizable parasitic feeders), and hepatobiliary toxicities in the target region (e.g., biloma, biliary necrosis). On the other hand, after successful ss-cTACE, a new lesion fulfilling the HCC-hallmarks outside the TACE-treatment is not a progressive disease by mRECIST criteria, and thus could be treated with an additional ss-cTACE. To summarize, the panel agreed that patients should be treated with TACE until they are no longer considered suitable for TACE.

The expert panel

 − Recommends the use of immediate post-treatment MDCT or CBCT to assess tumor targeting.
 − Recommends the use of contrast-enhanced CT as initial imaging on first follow-up after ss-cTACE
 − Recommends the use of MRI if remaining tumor viability cannot be confidently assessed on CT
 − Acknowledges that if no response is obtained after two ss-cTACE sessions performed within six months, the patient must be considered TACE-refractory and proposed for alternative therapy.
 − Recommends that patients should be treated with TACE until they are considered TACE unsuitable.

## Conclusion

As a multidisciplinary group of experts in the treatment of HCC, we assessed the results of a thorough literature review regarding the treatment of unresectable HCC with TACE, to identify the main factors that could impact indications, technique, and outcomes of ss-cTACE. Our goal is to help optimize procedure standardization and level out variability across centers. This paper is in alignment with most recent staging and treatment allocation systems that separate the heterogeneous intermediate HCC population in 3 subgroups [5]. The subgroup allocated to TACE is a population with well-defined nodules, preserved portal flow, in whom selective vascular access to the tumors(s) is possible, which clearly corresponds to what we defined in this paper as the best candidates for ss-cTACE. Some emerging clinical data on new combined treatments, including TACE, might change treatment allocation in the population with more diffuse liver involvement that could be targeted with a combination or sequence of molecular targeted- or immune-therapies and TACE.

These recommendations from the Expert meeting have been drafted to encourage treatment standardization in HCC patients, including patient selection and ss-cTACE delivery. They may be used as a general guide, but the interventional radiologist treating the patient is ultimately responsible for the treatment approach. Referring physicians must be aware of the treatment selected, with all possible complications, adverse effects, and post-treatment care. Finally, patients are best served by multidisciplinary decision-making, and interventional radiologists should take an active role in patient selection, treatment allocation, and post-procedural care.
